# Molecular evidence of *Ureaplasma urealyticum *and *Ureaplasma parvum *colonization in preterm infants during respiratory distress syndrome

**DOI:** 10.1186/1471-2334-6-166

**Published:** 2006-11-21

**Authors:** Rosario Cultrera, Silva Seraceni, Rossella Germani, Carlo Contini

**Affiliations:** 1 Infectious Diseases, Department of Clinical and Experimental Medicine, University of Ferrara, Ferrara, Italy; 2 Pediatric and Neonatal Pathology Unit, "G. Salvini" Hospital, Corso Europa, Rho, Milan, Italy

## Abstract

**Background:**

*Ureaplasma urealyticum *and *U. parvum *have been associated with respiratory diseases in premature newborns, but their role in the pathogenesis of the respiratory distress syndrome (RDS) is unclear. The aim of this study was to detect, using molecular techniques, the role of *Mycoplasma *spp. and *Ureaplasma *spp. in respiratory secretion and blood specimens of preterm newborns with or without RDS and to evaluate the prevalence of perinatal *U. urealyticum *or *U. parvum *infection. The influence of chemotherapy on the clinical course was also evaluated.

**Methods:**

Tracheal aspirate or nasopharingeal fluid samples from 50 preterm babies with (24) or without RDS (26) were analysed for detection of *U. urealyticum *and *U. parvum *by culture identification assay and PCR. Sequencing analysis of amplicons allowed us to verify the specificity of methods. Clarithromycin (10 mg kg^-1 ^twice a day) was administered in ureaplasma-positive patients who presented clinical signs of RDS.

**Results:**

15/24 neonates with RDS (p < 0.001) and 4/26 without RDS were found PCR-positive for *U. urealyticum *or *U. parvum*. Culture identification assay was positive in 5/50 newborns, three of which with RDS. Sequencing analyses confirmed the specificity of these methods. Association of patent ductus arteriosus with ureaplasma colonization was more statistically significant (p = 0.0004) in patients with RDS than in those without RDS.

**Conclusion:**

Colonization of the lower respiratory tract by *Ureaplasma *spp. and particularly by *U. parvum *in preterm newborns was related to RDS. The routine use of molecular methods could be useful to screen candidate babies for etiologic therapy.

## Background

Respiratory distress syndrome (RDS), a major cause of morbidity and mortality in preterm infants, is caused by cardiopulmonary immaturity with a deficiency of surfactant in the alveolar space. RDS is often associated with patent ductus arteriosus (PDA), intraventricular haemorrhage and chronic lung diseases which could complicate RDS [1,2]. Among the infectious agents that colonise or infect the genital and the lower urinary tract [3,4], *Ureaplasma urealyticum *and *Mycoplasma hominis *have been linked to the development of chronic lung diseases (CLD) [5,6] or brochopulmonary dysplasia [7,8], while their role in the pathogenesis of RDS remains controversial. Several lines of evidence suggest that *U. urealyticum *may cause lung injury through a number of mechanisms including the inhibition of pulmonary surfactant by phospholipase A2 produced by *U. urealyticum *and the production of interleukins as well as of soluble intercellular adhesion molecules [9,6].

*U. urealyticum *and *Mycoplasma *are among the less frequently diagnosed respiratory pathogens in a clinical environment, mainly because of the lack of standardised and specific diagnostic tests. PCR techniques have proved useful in detecting *U. urealyticum *in clinical specimens [10,11] due to time saving and the possibility of directly identifying the mycoplasma species. In addition, studies of genome sequencing of *U. urealyticum *have allowed two different species to be distinguished, serotyped and genotyped as *U. urealyticum *(Shepard *et al*. 1974, *species*; biovar 2; serovars 2, 4, 5, 7–13) and *U. parvum *(Robertson *et al*. 2002, sp. nov.; biovar 1; serovars 1, 3, 6, 14) [12,13].

Accumulating data suggest a possible link between *U. urealyticum *and the clinical chronic lung diseases outcome. Recently, Kotecha and co-workers have described the colonization of their study patients with either biovar 1 or biovar 2, but not both, without a different distribution inside patient groups [6]. The role that human ureaplasmas could have in the pathogenesis of RDS in preterm newborns is not completely known. The distinction of *U. urealyticum *and *U. parvum *species could also open new perspectives of study.

The aim of this study was to evaluate the prevalence and the short-term outcome of perinatal *U. urealyticum *and *U. parvum *infections in premature infants by molecular approaches including species-specific PCR assays, compared with a test for the rapid identification of *M. hominis *and *Ureaplasma *spp., in order to demonstrate an association between the colonization of these microrganisms and the development of RDS. Sequence analysis of the PCR products was also used to confirm two different *Ureaplasma *spp.

## Methods

### 1. Patients

Our population study included premature and full-term newborns who were admitted within 24 h after birth to the neonatal intensive care unit (NICU) of "G. Salvini" Hospital in the Rho District, Milan, Italy, between February 2001 and January 2002. Criteria of recruitment were i) the need for assisted ventilation with or without very low birth weight and the gestational age < 37 weeks and *ii) *the exclusion of major congenital abnormalities and of intrauterine transmitted infections, including TORCH, apart from mycoplasmal and ureaplasmal infections. Ventilatory requirements (on days 1, 7, 14, 21 and 28 of life) were summarized using the fractional inspired oxygen (F_i_O_2_), as the amount of oxygen delivered to the patient [1,7]. Mechanical ventilation was obtained by tracheal intubation or by nasal continuous positive airways pressure. Gestational age was established by the last normal menstrual period and ultrasound examination before 20 weeks of gestation. Data of maternal risk factors (hypertension, premature rupture of membranes [PROM], amnionitis, chorioamnionitis and antenatal steroids administration) were obtained from clinical charts. Mothers of the study children represented all socio-economic levels.

Seven patients were excluded from the study: four, because their gestational age was over 37 weeks, in spite of the need for ventilatory assistance, and three, because they did not need respiratory assistance and were transferred early to another clinic. According to recruitment criteria, infants admitted in the study were classified in two groups: group 1 included all infants with RDS (24); group 2 included newborns without RDS which needed ventilatory assistance by continuous positive airways pressure for no longer than 48 hours (26). A control group represented by 20 healthy full-term newborns, born by vaginal delivery, without a complicated course of pregnancy or clinical signs of respiratory diseases, was included in this study.

The infants were cared for according to the hospital ward's general principles concerning therapy, antibiotics, and ventilatory therapy. Patients were hospitalised until their clinical improvement and for not less than 28 days. Written informed consent was obtained from parents by attending physicians before the inclusion in this study, which was approved by the Regional Ethics Committee of the University of Ferrara. Research was carried out according to the Helsinki Declaration and its successive amendments.

Mothers presenting PROM received i.v. amoxycillin 1 g three times a day. Antenatal steroid treatment (betamethasone 12 mg twice a day) was given in 22 cases in which preterm delivery was anticipated at less than 34 weeks of gestation. Vaginal specimens were obtained at the time of the patient's admission for being in labor. Cotton – swab samples were collected from the urethra and the endocervix for identification of *M. hominis *and *Ureaplasma *spp.; the swabs were harvested in the transport medium supplied by the manufacturer and analysed by a culture identification assay (Mycoplasma Duo test kit, Bio-Rad Laboratories, Italy).

All newborns with umbilical venous catheter received i.v. ampicillin mg 100 kg^-1 ^plus netilmicin 2.5 mg kg^-1^, for at least 72 hours and until the results of cultures were available. Plasmatic dosage of netilmicine was made every 4–5 days. Echocardiographic studies were performed in each very low birth weight infant and in patients presenting clinical signs consistent with PDA and RDS. 11 newborns received dexamethasone i.v. administration (0.25 mg kg^-1 ^every 12 h for 3 days) in order to improve pulmonary function and to prevent the development of CLD. The shorts courses of glucocorticoids were repeated every 10 days until mechanical ventilation and oxygen-therapy were suspended. Patients with evidence of RDS received endotracheal treatment with natural porcine surfactant (Curosurf, Chiesi, Italy) (200 mg kg^-1 ^for the first dose, 100 mg kg^-1 ^for the following doses, with a minimal interval of 8 hours). Newborns that resulted positive for *U. urealyticum *or *U. parvum *by microbiological or PCR analyses received intravenous (i.v.) clarithromycin at a dosage of 10 mg kg^-1 ^twice a day. Clinical specimens of patients with or without clarithromycin treatment were tested before the tracheal intubation was removed or after clinical improvement, respectively.

### 2. Disease definition

RDS was defined by the presence of 2 or more of the following criteria: evidence of respiratory compromise shortly after delivery (tachypnea, intercostal retractions, expiratory grunting) [2], and a persistent oxygen requirement for more than 24 hours (F_i_O_2 _> 0.4), administration of exogenous pulmonary surfactant, and/or radiographic evidence of hyaline membrane disease [1,2,14]. Chest radiographs were obtained as part of routine clinical care, and their interpretation was performed by a single radiologist, who was unaware of the clinical data and culture results. CLD was defined as the need for supplemental oxygen at 28 days of age or the need for supplemental oxygen at 36 weeks postconceptional age [15].

PDA was diagnosed by Doppler echocardiographic studies. Ibuprofen lysine (10 mg kg^-1 ^for the first day, followed by 5 mg kg^-1 ^after 24 and 48 hours) was intravenously given to infants for the treatment of PDA. After medical treatment an echocardiographic study was performed to confirm that the ductus arteriosus was closed [16]. In one patient, after the failure of medical treatment with ibuprofen and then with indomethacin, surgical ligation was performed.

### 3. Clinical specimens

Endotracheal aspirates (TA) or nasopharyngeal fluids (NF), from intubated or non-intubated infants, respectively, and serum samples were collected soon after the birth, within the first 12 hours of life and before administration of surfactant or antibiotics, in order to exclude nosocomial transmission of infectious agents and to avoid any possible interference with laboratory findings. After mechanical suction, TA and NF, collected by catheters, were inoculated in 2 mL of an adapted transport media supplied by the manufacturer and then analysed by culture identification assay. 40–50 μL of each sample diluted in the transport medium were placed in plates containing a specific medium to identify *U. urealyticum/U. parvum *and *M. hominis*, distinguished by urea or arginine utilization, respectively. Blood samples were inoculated into a blood-culture bottle for either aerobic or anaerobic bacteria (1 mL) and incubated at 37°C for 24 and 48 hours. Positive cultures were further inoculated into specific media to identify the bacteria species. Sterile cotton swabs were rubbed over the infant's posterior pharynx, over the ear and rectum and cultured for conventional bacteria twice weekly until discharge, as part of routine surveillance in the NICU.

### 4. Polymerase chain reaction

We employed the common careful procedures (aliquoting autoclaved reagents, UV-irradiation treatment for surface laboratory benches, filter-tips and adding DNA last) in order to avoid the risk of contamination by DNA or PCR product carryover.

Each TA or NF specimen (approximately 5 to 10 mL) was collected in ureaplasma broth, containing urea, ampicillin 66 mg mL^-1 ^and foetal calf serum 20% (A3 medium), and in *M. hominis *broth, containing arginine, ampicillin 66 mg mL^-1 ^and foetal calf serum 20% (BDA medium). 2 mL of each sample was then added to an equal volume of phosphate buffered saline (PBS, pH 7.2) and centrifuged at 12,000 g for 15 min. A portion of pellet was resuspended in 1 mL A3 or BDA medium and stored until required for PCR evaluation. Sample lysis was done as previously described on 200 μL of each sample. Before PCR, 2 μL direct and tenfold dilutions of each sample were amplified in order to test that lysis had occurred and to identify the possible inhibition of amplification due to the presence of contaminants in the sample [17]. Specimens were formerly screened by *Mycoplasma *spp. and *Ureaplasma *spp. specific primers (MGSO/GPO1, 717 bp) and then by species-specific primer pairs for *U. urealyticum *and *U. parvum*, and *M. hominis*. In order to investigate other most common human mycoplasmas, PCR assaywas used to search for *M. pneumoniae *and *M. genitalium*. Genus and species-specific mycoplasma oligonucleotide primers (Genset, France) were deduced from either the conserved or variable 16S RNA regions, while UU3, UU4 and UU5 *U. urealyticum *primers were also obtained from the sequence of urease gene (table 1) [11,17]. PCR assay to distinguish *U. urealyticum *and *U. parvum *was performed by employing oligonucleotides previously described [18]. Amplification conditions for the different PCR systems are summarized in table 1. MGSO/RNA5 primer pairs were less specific and more sensitive than GPO1/MGSO. A true positivity was considered if obtained with both pairs of primers. The thermal cycler apparatus was a PCR-Express PCYL001 (Hybaid, UK).

**Table 1:** Oligonucleotides employed as primers in PCR assays for detection of *Mycoplasma *and *Ureaplasma *species.

Species	Primers name (sense/antisense)	Gene targets, amplicon size (bp)	Conditions of n-PCR amplification
*Mycoplasma *spp. [17]	RNA5 (agagtttgatcctggctcagga)/MGSO (tgcaccatctgtcactctgttaacctc)	16S rRNA, 1005	1 cycle of 15 min. at 95°C; 30 cycles of 30 sec. at 95°C, 90 sec. at 58°C, 90 sec. plus 1 sec./cycle at 72°C; final extension of 10 min. at 72°C.
*Mycoplasma *spp. [17]	GPO1(actcctacgggaggcagcagta)/MGSO (tgcaccatctgtcactctgttaacctc)	16S rRNA,717	
*M. pneumoniae + M. genitalium *[17]	PNEU+GEN (cctgcaagggttcgttattt)/MGSO (tgcaccatctgtcactctgttaacctc)	16S rRNA,851	
*M. hominis *[17]	HOM (tgaaaggcgctgtaaggcgc)/UNI^- ^(taatcctgtttgctccccac)	16S rRNA,589	
*U. urealyticum + U. parvum *[11]	UU3 (gatggtaagttagttgctgag)/UU4 (acgacgtccataagcaact)	Urease, 456	0.8 μM of each primer, MgCl_2 _1.5 mM; dNTP 0.2 mM; *Taq *DNA polymerase 1U/50 μl.
*U. urealyticum + U. parvum *[11]	UU5 (caatctgctcgtgaagtattac)/UU4 (acgacgtccataagcaact)	Urease, 429	

*U. urealyticum *[18]	U8 (gaagatgtagaaagtcgcgtttgc)/P6 (ggtagggataccttgttacgact)	16S rRNA, 1312	1 cycle of 5 min. at 95°C; 30 cycles of 30 sec. at 95°C, 30 sec. at 58°C, 2 min 30 sec. at 72°C.
*U. parvum *[18]	U3 (tagaagtcgctctttgtgg)/P6 (ggtagggataccttgttacgact)	16S rRNA, 1305	1 μM of each primer, MgCl_2 _1.5 mM; dNTP 0.2 mM; *Taq *DNA polymerase 1.25U/50 μl.

20 μL of amplified products were electrophoresed on 0.8% agarose gels in TAE buffer together with a number of positive and negative controls and bands were visualised by ethidium bromide staining. Sensitivity of the PCR assay was defined at 10 fg of DNA corresponding to 10 – 15 mycoplasmas mL^-1 ^or 10 CFU.

### 5. Sequence analysis

The amplification products from TA and NF that resulted *Mycoplasma *spp. or *Ureaplasma *spp. positive were analysed by cycle sequencing (Ampli-Cycle Sequencing Kit, Perkin Elmer, CA, USA), in order to verify the specificity of employed primers and to identify the serovars of the *Ureaplasma *species. PCR products were purified from amplification reaction mixture and cycle sequencing was performed according to the manufacturer's instructions (Redivue™, Amersham Pharmacia Biotech, England).

In order to confirm the accuracy of the sequencing analysis, the same PCR products were also sequenced by an ABI PRISM^® ^377 DNA Sequencer. The closest matches for the sequences obtained were determined by BLAST searches [19]. Serovars of *U. parvum *or *U. urealyticum *were determined by identities detected with the BLAST search in the GenBank.

### 6. Statistical analysis

The medians for the gestational age, birth weight, days of hospitalisation, Apgar score and hours of mechanical ventilation were given. Comparison between frequencies of clinical parameters and molecular analyses in the examined groups was performed with Fisher's exact test. A logistic regression was made, correlating the independent variables, to calculate the statistical significance by maximum likelihood χ^2^-analysis. P-values < 0.05 were considered significant. *STATISTICA *software was used.

## Results

Fifty patients were admitted in the study, 24 in group 1 and 26 in group 2. The most relevant clinical features of patients are shown in table 2. Two pairs of twins were included in the study population, one born by vaginal delivery and the other by Caesarean delivery. One baby in each pair showed typical clinical signs of RDS and was therefore included in group 1.

**Table 2:** Characteristics of the examined population stratified by group of patients with or without RDS.

	**GROUP 1 (n. 24)**			**GROUP 2 (n. 26) **^**§**^	
	
	*U. urealyticum *positive PCR (n. 5)	*U. parvum *positive PCR (n. 10)	Ureaplasma negative PCR (n. 9)	*U. urealyticum *positive PCR (n. 4)	Ureaplasma negative PCR (n. 22)
Culture identification assay positivity (%)	1 (20)	2 (20)	0	2 (50)	0
Median gestational age, weeks (range)	35+3 (28; 37+5)	31+2 (23+2; 35+2)	32+5 (27+2; 37)	32 (30+5; 34)	32+4 (30; 37)
Median birthweight, g (range)	2530 (1070–3000)	1660 (720–2950)	1930 (1030–2690)	1340 (1140–2260)	1635 (750–3580)
Median days of hospitalisation	30 (22–60)	33 (7*–60)	22 (10–70)	32 (18–42)	23 (4–60)
Median Apgar score, 1' (range)	8 (6–10)	8 (5–10)	4.5 (1–10)	9.5 (8–10)	7 (1–10)
Median Apgar score, 5' (range)	9 (8–10)	9.5 (8–10)	10 (3–10)	9.5 (9–10)	10 (6–10)
Gender, male (%)	1 (20)	3 (30)	2 (22.2)	3 (75)	6 (27.3)
Antenatal steroids (%)	2 (40)	8 (80)	2 (22.2)	3 (75)	8 (36.4)
Antenatal ampicillin (%)	2 (40)	7 (70)	3 (33.3)	2 (50)	6 (27.3)
PROM > 12 hours (%)	0	2 (20)	0	1 (25)	4 (18.2)
Caesarean delivery (%)	3 (60)	5 (50)	6 (66.7)	2(50)	13 (59.1)
Blood culture positive (%)	1 (6.7)	0	1 (11.1)	0	0
Surfactant (%)	5 (100)	10 (100)	5 (55.6)	0	0
PDA (%)	4 (80)	10 (100)	5 (55.6)	1 (25)	7 (31.8)
Neonatal dexamethasone (%)	6 (40)		2 (22.2)	1 (25)	2 (9.1)
Mechanical ventilatory requirement (%), median duration (hours)	5 (100), 60.5	9 (100), 72	6 (66.7), 72	3 (75), 60	4 (18.2), 48
F_i_O_2 _> 0.40 (range)	5 (0.40)	9 (0.40–1.00)	5 (0.40–0.90)	0	0
CLD at 28 days (%)	1 (20)	1 (10)	0	1 (25)	0
CLD at 36 weeks (%)	0	0	0	0	0

* patient died; ^§ ^no patient resulted *U. parvum *PCR positive in this group.

Culture identification assays showed five (10%) positive cases out of 50 patients, three of which with RDS, compared to 19/50 (38%; p < 0.05) obtained by PCR technique. Culture identification assays resulted negative for *M. hominis*. Vaginal cotton-swab specimens resulted positive by culture identification assay for *Ureaplasma *spp. either in 12 mothers of newborns with RDS or in 6 mothers of babies without RDS. 12/18 positive women gave birth to a newborn PCR positive for *U. urealyticum *or *U. parvum*, and 2/18 gave birth to a pair of twins.

**Figure 1:**
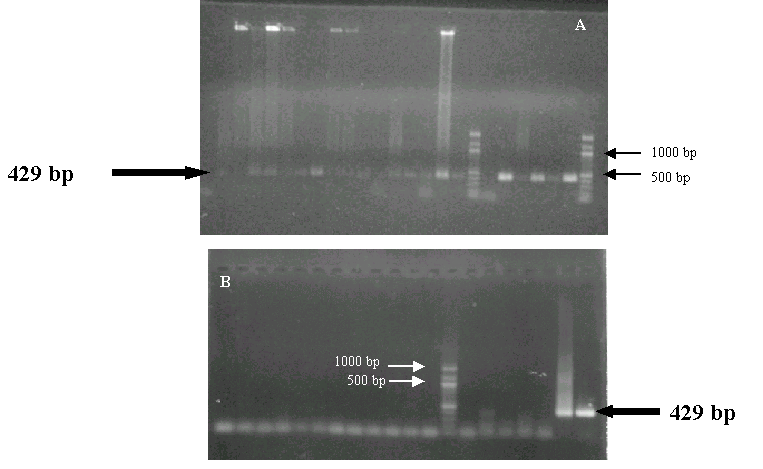
**Agarose gel electrophoresis of urease gene PCR**. 20 μL of amplified products for the urease gene were electrophoresed on 0.8% agarose gel in TAE buffer (40 mM tris-acetate, 1 mM EDTA, pH 8). A) Lanes 1–14: PCR results of TA samples of newborns with RDS; lanes 15–16: *U. urealyticum *and *U. parvum *PCR positive control; lanes 17 and 24: 100 bp ladder; lanes 18–23 PCR results of TA or NF samples of newborns without RDS. B: PCR negativity for *Ureaplasma *spp. of respiratory secretions from the control group; lane 13: 100 bp ladder; lanes 19–20: *U. urealyticum *and *U. parvum *PCR positive control.

Genus- and species-specific PCR demonstrated *U. urealyticum *and *U. parvum *DNA in 14/24 TA and 1/24 serum from the patients (62.5%; χ^2^-test: p = 0.0004) in group 1, and in 4/26 NF (15.4%) from the patients in group 2 (figure 1). *U. parvum *specific-PCR with pairs of primers U3/P6 and U8/P6 resulted positive in 10/15 patients in group 1. In particular, the patient with RDS and the identification of *U. parvum *DNA, died on the 8^th ^day of extrauterine life, due to complicated pneumothorax, intraventricular haemorrhage (grade III) and *Staphylococcus epidermidis *sepsis. This baby's respiratory secretions were not available. Of the two pairs of twins, two patients with RDS had a positive PCR for *U. parvum *only. No amplification signal was found for other mycoplasmas. NF samples from the newborns of the control group (n. 20) resulted negative with both culture identification and PCR assays.

One fact of interest is that a female with low birth weight and clinical signs of RDS, was admitted to NICU on two different occasions. During the first admission (45 days), NF samples resulted both culture identification assay and PCR negative. Fifteen days after discharge, she was admitted to NICU a 2^nd ^time, because of hypothermia and dyspnea. Ventilatory assistance was started because of a fine miliary mottling through the lungs at RX with an air bronchogram and a F_i_O_2 _at 0.35. NF samples collected during this re-admission, were PCR positive for *U. parvum*.

The detection rate of *U. urealyticum *and *U. parvum *was higher in TA of patients that received surfactant than in specimens of those not treated, with a high ureaplasmal positivity in the patients of group 1 (100%; χ^2^-test: p = 0.004). Moreover, there was a significant statistical difference in PCR results when compared with mechanical ventilatory requirements (100%; χ^2^-test: p = 0.004). The incidence of PDA was higher and statistically significant in patients in group 1, in comparison with those of group 2 (χ^2^-test: p = 0.04) (table 2), with a high *U. urealyticum *or *U. parvum*-PCR positivity detection rate which was higher in group 1 (n = 14; 93,3%) than in group 2 (n = 1; 25%). Gestational age and birth weight were not statistically significant for small amount of data.

**Figure 2:**
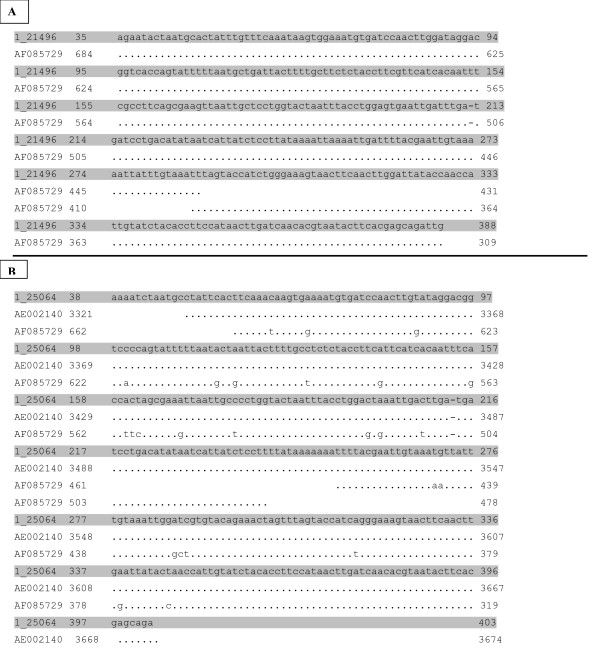
**BLAST sequence analysis of *Ureaplasma *spp. ureasegene amplicons**. BLAST sequence analysis of urease gene amplified fragments of *U. urealyticum *(GenBank AE002140) and *U. parvum *(GenBank AF085729) in two different respiratory specimens: sample SR1 (A) resulted identical to *U. parvum *and sample SR42 (B) resulted more identical to *U. urealyticum *than *U. parvum*. Nucleotide differences between sequences of two species are shown.

**Figure 3:**
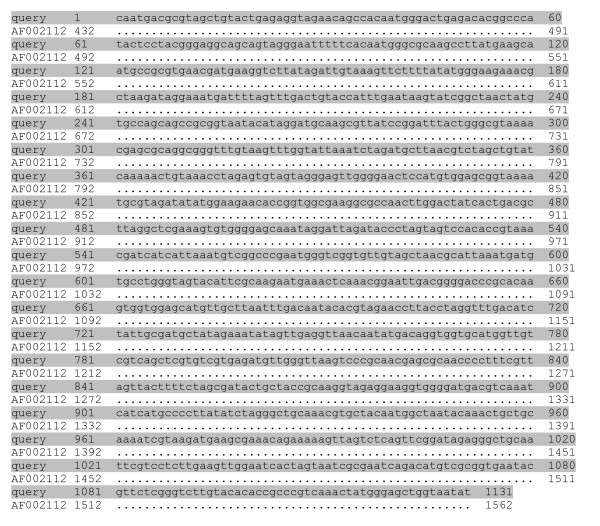
**BLAST sequence analysis of *Ureaplasma parvum *16SrRNA amplicons**. BLAST sequence analysis of 16S rRNA gene amplified fragments of *U. parvum *(GenBank AF002112) employing U3/P6 primers, in the sample SR1 resulted identical to *U. parvum*. Nucleotide differences between sequences of two species are shown.

**Figure 4:**
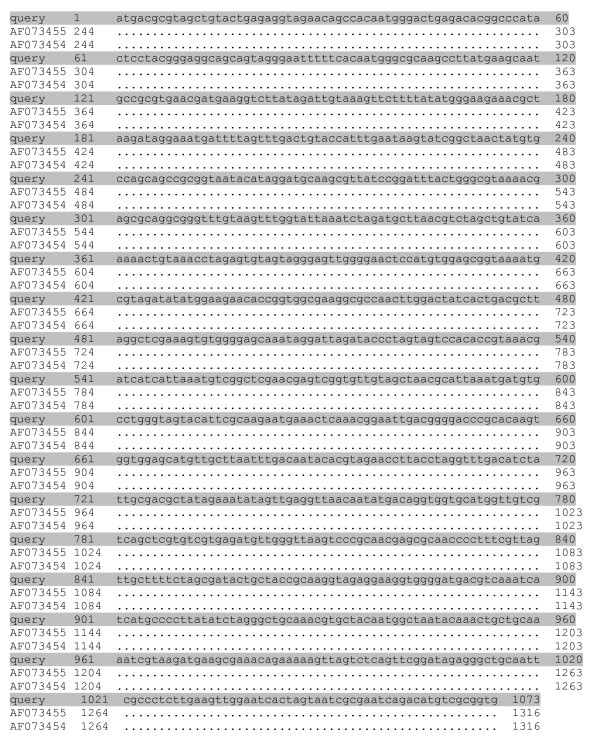
**BLAST sequence analysis of *Ureaplasma urealyticum *16S rRNA amplicons**. BLAST sequence analysis of 16S rRNA gene amplified fragments of *U. urealyticum *(GenBank AF073455, AF073454) employing U8/P6 primers in the sample SR42 resulted identical to *U. urealyticum*. Nucleotide differences between sequences of two species are shown.

BLAST sequencing analyses showed that amplified fragments of 456 bp (UU3/UU4) and 429 bp (UU4/UU5) aligned in the GenBank database with sequences of urease gene of *U. urealyticum *(GenBank AE002140) and *U. parvum *(GenBank AF0875729) (figure 2). DNA sequences of the 456 bp and 429 bp PCR amplicons from the urease gene of the isolates were 93.1–99.7 % identical to urease gene sequences for the different ureaplasma type strains. A more specific analysis by pairing of amplification sequences, confirmed that the amplicons were consistent with *U. parvum *in 10/15 patients, and *U. urealyticum *in 5/15 patients in group 1, and *U. urealyticum *in 4/4 patients in group 2 (χ^2^-test: p = 0.004). BLAST alignment of the 16S rRNA amplicons, obtained by PCR with U3/P6 or U8/P6 primers, confirmed the distinction of species and serovars evidenced by urease gene amplification (*U. parvum*: GenBank AF002112 [figure 3]; *U. urealyticum*: GenBank AF073455, AF073454 [figure 4]). All serovars of *U. parvum *were represented and serovars 2, 4, 8 and 13 were within the *U. urealyticum *species.

Patients that had a positive PCR assay for *U. urealyticum *and *U. parvum *were treated with intravenous clarithromycin at a dosage of 10 mg kg^-1 ^twice a day for ten days or until clinical improvement. These patients did improve clinically and respiratory secretions did result negative with laboratory assays. PCR positive TA or NF specimens were PCR negative after treatment. The follow-up of newborns showed clinical and laboratory signs for CLD after 28 days, in two patients in group 1, who resulted PCR positive *for U. parvum *and *U. urealyticum*, respectively, and in one patient in group 2, PCR positive *for U. urealyticum *only.

## Discussion

The aim of this study was to investigate the colonization of the respiratory tract of preterm newborns by *U. urealyticum, U. parvum *and *M. hominis *using an in-house PCR and to prove a possible association between RDS and ureaplasma infection. *U. urealyticum *and *M. hominis *were identified more frequently in respiratory secretions and less frequently in cerebrospinal fluid and blood specimens of preterm newborns with pneumonia, meningitis and sepsis, respectively [12,20]. A number of studies attempted to relate *U. urealyticum *colonization to the development of respiratory diseases in high risk newborns (gestational age < 28 weeks) and mostly with CLD, but the effective role of *U. urealyticum *and *U. parvum *in the pathogenesis of RDS when associated with prematurity remains controversial. A recent study showed an association of respiratory failure due to RDS with *U. urealyticum *colonization on bronchoalveolar lavage samples [6] using specific primers for *U. urealyticum *urease-gene, different from those employed by us.

After an accurate search of the biomedical literature by PubMed database [19], our study was the first work in which a PCR method was applied to distinguish *U. urealyticum *from *U. parvum *by species – specific primers directly on clinical respiratory samples of premature infants. Using this method, *U. parvum *was detected only in TA of patients with RDS and *U. urealyticum *in respiratory samples of both groups of patients, but no sample was colonized with both *Ureaplasma *spp. A significantly high difference in PCR results for *U. urealyticum *and *U. parvum *was detected especially in TA of newborns in group 1 that received surfactant by endotracheal instillation. A previous study supported the efficacy of exogenous surfactant administration in premature infants with RDS [15] and a recent finding, obtained using a culture based method, showed that 73% of preterm neonates colonized from *U. urealyticum *and *U. parvum *had RDS [21]. Another study has shown a prevalence (76–81%) of *U. parvum *in amniotic fluid from preterm complicated gestation [22]. Our findings suggested that *U. parvum *may play a more effective role in the pathogenesis of RDS than *U. urealyticum*.

Although in our study antibiotic therapy was restricted to newborns that resulted PCR positive for *Ureaplasma *spp. without a placebo-treated control group, the improvement of the clinical outcome observed in patients after clarithromycin treatment may be a further suggestion that the colonization of the respiratory tract by *U. urealyticum *and *U. parvum *could be considered an important factor that contributes to acute lung injury. Moreover, as shown by culture and PCR DNA detection, this treatment led to eradication of *Ureaplasma *spp. in TAs as well as in NF specimens, before the removal of tracheal intubation.

The PCR assay showed a greater sensitivity than culture identification assay in detecting *Ureaplasma *spp. At the time the samples were collected, culture identification assay, recently approved only for testing genital specimens with a good sensitivity, was also applied to respiratory secretions.

A body of evidence suggests that *Ureaplasma *spp. infections, as well as genital infections, are linked to complicated pregnancy (PROM, chorioamnionitis, Caesarean delivery or funisitis) or neonatal outcome (low birth weight, gestational age < 37 weeks, neonatal sepsis, CLD, BPD) [23,24] but infants born premature by mothers with chorioamnionitis did not develop RDS despite the higher association of ureaplasma biovar 1 (*U. parvum*) colonisation with CLD [15]. As regards the clinical parameters of the pregnancy, apart from longer hospitalisation in newborns found positive for ureaplasma, compared with those negative with both methods (table 2), we did not find statistical differences between the two groups for small amount of our data, according to previous study [22]. Our study was specifically focused on infants with RDS who did not have any clinical or laboratory evidence of infection at birth; thus suggesting that *U. urealyticum *or *U. parvum *must be considered important cofactors to the development or initiation of acute lung injury, according to other studies [6,25,26]. Interestingly, *U. parvum *PCR positivity was detected in TAs of twins with RDS (one of each pair of twins enrolled in this study) but not in the respective siblings without RDS. These data could indicate that ureaplasmal infection may be a possible causative factor in the pathogenesis of RDS. No link with the method of delivery as a favourable factor was found, although our data are not statistically significant.

A further consideration concerns PDA and ureaplasma colonization. Statistical analysis has shown that the incidence of PDA in the two groups of patients was weakly significant but a greater statistical significance was observed in patients with PDA and RDS that resulted positive for *U. urealyticum *or *U. parvum*. These data were consistent with a previous study which has demonstrated that PDA may be considered a pathology influencing respiratory morbidity [27]. These findings contributed to providing further evidence that *U. parvum *and *U. urealyticum *colonizations could complicate the clinical course of RDS in preterm infants with PDA.

Another finding of this study was the positivity of *U. parvum *in the female patient during the 2^nd ^admittance, at two months of extrauterine life, compared with the PCR-negativity found at the 1^st ^admission. We suppose that ureaplasmal load of the first NF sample was very low, so that *U. parvum *was undetectable. Its persistence in the lower respiratory tract following the first discharge as demonstrated by PCR positivity, supported a replication of ureaplasmas which may lead to histological lung damage, as shown by the clinical picture of RDS. In this case, our data agreed with those of Castro-Alcaraz and co-workers that described three patterns of *U. urealyticum *colonization of preterm infants developing a CLD: persistent positive, transient and late transient colonization [28]. The improvement of the clinical picture after clarithromicyn treatment supported this hypothesis.

The role of the immune response in determining RDS is not secondary. *In vitro *and *in vivo *studies demonstrated a release of proinflammatory cytokines (TNF-α, IL-1β, IL-6) and G-CSF and GM-CSF from lymphomonocyte and alveolar cells, respectively, stimulated by mycoplasma- and ureaplasma-derived lipoproteins [29,30]. Biochemical mechanisms, such as the inhibition of pulmonary surfactant production by phopspholipase A_2 _[9] may result in acute lung damage.

## Conclusion

*U. parvum *more than *U. urealyticum *were found by PCR in respiratory secretions of preterm newborns with RDS, indicating a possible role of these *Mollicutes *in the pathogenesis of RDS. Our findings support the evidence that PCR could be a highly sensitive and specific technique for detecting *Ureaplasma *and for distinguishing *U. urealyticum *from *U. parvum *directly in clinical specimens, suggesting that the described PCR methods could be considered as a possible true "gold standard", because of the selection of primers with wide specificity that react with the DNA of *Ureaplasma *or *Mycoplasma *derived from target sequences in the highly conserved regions of the genes. The routine use of this technique for NICU patients could have a role in more accurately diagnosing infection by *U. parvum *and *U. urealyticum*, especially in newborns at risk of developing RDS.

## Competing interests

The authors declare there are no competing financial or non-financial interests (political, personal, religious, ideological, academic, intellectual, commercial or any other) in relation to this manuscript.

## Authors' contributions

RC conceived this study, carried out the molecular genetic studies, participated in the sequence alignment, performed the statistical analysis and drafted the manuscript. SS participated in the molecular analyses and sequence alignment. RG carried out the clinical study and participated in the design of the study. CC participated in the design of this study and its coordination and helped to draft the manuscript. All authors read and approved the final manuscript.

*All Authors contributed to the writing of the final manuscript*.

*All Authors read and approved the final manuscript*.

## Pre-publication history

The pre-publication history for this paper can be accessed here:


